# Is there gender inequality in the impacts of energy poverty on health?

**DOI:** 10.3389/fpubh.2022.986548

**Published:** 2022-12-12

**Authors:** Ziyu Zhang, Yuting Linghu, Xue Meng, Hong Yi

**Affiliations:** ^1^College of Big Data Application and Economics, Guizhou University of Finance and Economics, Guiyang, China; ^2^College of Business Administration, Guizhou University of Finance and Economics, Guiyang, China

**Keywords:** energy poverty, intra-household division of labor, moderating effects, status inequality, gender inequality

## Abstract

This paper aims to study the gender inequality in the health impacts of energy poverty. Using the Chinese Family Panel Studies (CFPS) 2018 data, we examine the gender heterogeneous effects of energy poverty on residents' health. The empirical results indicate that energy poverty would increase the ratio of respiratory disease, hospitalization as well as the healthcare expenditure. The effects are moderated by housework time. Moreover, we explore the gender inequality of the health impacts and find that women's health is more severely impaired and the effect of gender inequality is heterogeneous between urban and rural areas. We further investigate the historical origins of intra-household division of labor and reveal that the root of gender inequality in the health effects of energy poverty is status inequality. The government should give the first priority to energy poverty alleviation through modern energy network construction. Providing the energy-deprived families with ventilation equipment and medical insurance should be considered as the next step. Finally, it is imperative to eradicate urban-rural dual structure and legislate to ensure gender equality in the whole society.

## Introduction

Access to clean energy is not only indispensable to the human daily life but also necessary to people's health. According to International Energy Agency (IEA), despite extraordinary progress during the last two decades, more than 2.5 billion people lack access to clean energy for cooking worldwide, air pollution induced by the consumption of solid fuels and kerosene causes nearly 2.5 million premature deaths from illness every year in 2021 (1). Consequently, the health impacts of energy poverty have raised significant interest amongst academics and policymakers, especially in less developed countries.

Indoor air pollution induced by cooking, heating and other household activities has severe implications for human health. For the energy poor, the inhalation frequency of toxic gases and particles increases with the number of hours spending on housework, which results in severer health hazards. Given that women tend to spend more time on housework especially in traditional agricultural countries (2, 3), they are more likely to suffer from indoor air pollution and breathe in pollutants produced by solid fuel combustion. As a result, for energy poor families, women's health tends to be more severely affected than men's. In other words, there may be gender inequity in the health impacts of energy poverty. Although a plethora of literature has investigated the link between energy deprivation and health (4, 5), to our knowledge, little attention has been devoted to the gender gap in the health impacts of energy poverty.

As the biggest developing country, China has made tremendous progress in alleviating energy poverty, especially achieved 100% electrification in 2014. Yet there still have been almost 28.7% of people in China primarily using solid fuels for basic energy needs like cooking and indoor heating for the sake of costs or inadequate related equipment by 2019, accounting for 16.07% of the global energy poor (1). Moreover, China is also the largest transitional country with urban-rural dual structure—urban areas are industrial societies while rural areas remain agricultural societies, providing an excellent sample for studying gender inequality in the health impacts of energy poverty across sectors. In addition, the social concepts of gender equality has also been popularized in China with the remarkable development of the economy.

Consequently, investigating the gender inequality in terms of health impacts of energy poverty in China is imperative. First, investigating the heterogeneous health impacts of energy poverty between men and women in the largest developing country will facilitate global energy poverty alleviation and gender equality. Second, evidence from China can shed light on the policy-making for both industrial and agricultural countries throughout the world due to urban–rural dual structure. Additionally, findings from China will also provide lessons for countries that are undergoing rapid economic transformation.

Using a unique data set based on a survey applied to 27,407 individuals across the country in 2018, this paper aims to fill the gap in the existing literature by studying the gender inequality in the health impacts of energy poverty and examining moderating effects of housework. Furthermore, this paper researches the gender inequality in terms of health impacts of energy poverty and analyzes the sector and age heterogeneity. In addition, we also explore the historical origins of gender inequality by tracing the root of the intra-household division of labor. The results will not only contribute to formulating policies to eradicate energy poverty and mitigate gender inequality but also facilitate the achievement of Sustainable Development Goals (SDGs)−17 goals to be achieved before 2030 by the United Nations (UN) based on sustainable development of humanity.

This paper contributes to existing literature on the relationship between energy poverty and health status in following aspects. First, we investigate the nexus between energy poverty and health from the perspective of gender inequality. The empirical results demonstrate that women's health is more severely jeopardized than men's, shedding light on the analysis of gender difference in related studies. Second, by examining the moderating effects of housework time, we point out that the adverse effects of energy poverty on health are exacerbated by housework time, offering a new explanation how gender discrimination causes health inequality between men and women. Third, we study the heterogeneous effects of energy poverty on health across sectors and ages. Our findings point out that the gender inequality of energy poverty on health is diminishing with urbanization, development of economy and progress of social concepts. Finally, this paper incorporates historical perspectives into the causality between health status and energy deprivation and reveals that gender inequality mentioned above is attributed to status inequality, providing a new insight of mitigating health hazards of energy poverty.

The remainder of this paper is organized as follows. Section Literature review reviews the relevant literature. Section Methodology describes dataset, variables, and statistical characteristics. Section Main Results reports the main empirical results. The last section summarizes the main conclusions and draws some policy implications. Article types.

## Literature review

### Energy poverty and health

In general, energy poverty is defined as inaccessibility or unaffordability to sustainable modern energy for cooking or warming up (5–10). Solid fuels such as coal, straw and firewood widely used by energy-poor groups for cooking and heating generate air pollutants and particles that are harmful to health (11, 12). The above-mentioned implications can be aggravated in the absence of necessary ventilation equipment (13–15). More clearly, Ekholm et al. (16) noted that households living in rural India cook daily almost entirely with traditional biomass fuels, which may lead to premature deaths from respiratory, cardiovascular, and other diseases. Thereafter, some scholars further emphasized the health hazards of energy poverty (5, 17–19). In addition, Oum (20) and Churchill et al. (21) indicated that energy-poor households have a higher tendency to physical degeneration and excessive medical expenditure, and these circumstances may impair social wellbeing and the capability of improving living conditions.

Given that women tend to spend more time on domestic activities like cooking and cleaning, females in energy-deprived families are inevitably exposed to harmful compounds produced by solid fuel combustion (22, 23). Viegi et al. (24) found that attacks of shortness of breath accompanied by wheeze, dyspnea, and cardiovascular conditions in female non-smokers are associated with the use of stoves or forced-air circulation for heating in middle Italy. Torres-Duque et al. (25) pointed out that chronic obstructive pulmonary diseases in women are strongly linked with indoor biomass smoke, and lung cancer in women is clearly associated with household coal use. Smith-Sivertsen et al. (26) employed a random trial in Guatemala and found that using solid fuels for cooking is harmful to women's respiratory symptoms and lung functions. Abbas et al. (27) indicated that an empirically significant negative causal relationship has been found between the indicators of multidimensional energy poverty and health of women.

Despite the fact that energy poverty can endanger health status especially women's health, very little research has been conducted on gender inequality in terms of impacts of energy poverty on health. However, the gender heterogeneity of the interplay between energy poverty and health status plays a vital role in alleviating energy poverty and mitigating gender inequality.

### The moderating role of housework time

Considerable literature attributed gender inequality in health to economic status, social customs and law (28–30). Thanks to lower status in labor market, women's labor force participation is minimal or secondary to their domestic contributions (30, 31). As a result, there does exist gender discrimination with regard to intra-household resource allocation such as nutrition and medical care (32–34). Moreover, although social customs like dowry system would increase the stress for both men and women, men are inclined to have a lower likelihood of chronic illness (29). Additionally, Anderson (28) stressed that gender inequality can also be enhanced by weaker female marital property laws, which leads adult women (aged 15–49) three times more likely than men to be infected HIV on average. This alarming phenomenon is common in sub-Saharan Africa.

Due to disadvantages in labor market, women tend to be homeworkers and spend more energy and time domestic work (30, 31, 35–37). In this context, substantial literature about intra-household division of labor has shown that females tend to devote more time to household chores (3, 38–40). Ding and Chen (38) investigated the distribution of domestic activity time of rural inhabitants from poor areas in China. The results suggested that females spend up to 26 h a week on domestic works like firewood collection and cooking, which is much higher than 9 h of males. Moreover, they pointed out that cooking accounts for more than 90% of the time in household activities involving energy consumption, and women are responsible for cooking in most Chinese families. Similarly, using the data of 3,255 rural households collected by the China Health and Nutrition Survey (CHNS) from 2000 to 2011, Wei et al. (40) demonstrated that the share of adult females responsible for cooking is up to 80%, whereas <20% of adult males do that.

Notably, the impacts of energy poverty on health are positive with the extent of indoor air pollution. Considering that cooking accounts for more than 80% of housework time in countries such as China and Italy (25, 38, 41), indoor air pollution induced by cooking-dominant housework has become the primary source of health hazards in such countries. Moreover, Lim et al. (42) proposed that indoor air pollution due to using solid fuels for daily cooking is the third largest risk factor of disability adjusted life years and the most important environmental health risk factor as well. Cooking with poorly ventilated system and low combustion efficiency has adverse effects on the health of individuals, which equals to smoking two packs of cigarettes a day (43). In addition, Van Vliet et al. (44) conducted a study on rural areas in Ghana, which amplified that individuals exposed to indoor pollution caused by cooking are more likely to develop respiratory diseases like asthma than other individuals. The severity is strongly associated with the peak of pollution, which is consistent with the findings of Kurmi et al. (45) in Nepal.

While a relatively new body of literature has revealed that cook-dominant housework is the major source of health hazards, and women tend to spend more time on domestic affairs, little attention has been paid to examining the moderating effects of housework time on energy poverty and health status. Nonetheless, the moderating role of housework time is probably the direct reason that results in the gender inequality of the health impacts of energy poverty.

### Social status inequality and housework time

Regarding the reasons why females spend more time on housework than males, Boserup (2) emphasized that plowing agriculture requires sufficient power to operate plows and domesticate livestock, which enables males to gain a comparative advantage in economic activities and thus acquires a better economic position. In contrast, females are gradually marginalized and then turn to be responsible for less demanding but more elaborate housework (3). Even though advancement in technology has made agriculture increasingly independent on the muscle power of human beings, practically, it is usually males who learn to operate new equipment (46, 47). Moreover, Ding et al. (48) argued that the types of crop cultivation are the key factors affecting intra-household division of labor and the social status of females. In addition, several articles underscored the importance of cultural norms and legal systems (28, 29, 49–52). Overall, the position of males and females in agricultural economic activities has not fundamentally changed.

However, the uneven division of housework is not so prevalent until human being enters into industrial age. As the production mode and the economic position of females have differed from agricultural civilization (53, 54), a large number of married women are in the labor force and nearly equally share economic resources with their husbands. As a result, the husband's breadwinner role and the wife's housekeeper role do not retain their primary place within the family (55). In light of the fact that both husbands and wives are considered as breadwinners, the status of women would be undoubtedly on the rise and couples might share the housework relatively equal. In view of this, many scholars have argued that the traditional intra-household division mode—husbands' responsibility for outside work and wives' responsibility for domestic work—is gradually evolving into modern mode—relatively conjugal equal responsibility for outside and domestic work—with the passage of time (56–58).

Although previous literature has suggested that social status inequality is the root of the disproportionate division of housework time, to our knowledge, none of studies have explored the nexus between social status and the gender differences of the health hazards of energy poverty. Furthermore, if intra-household division of labor changes with the evolution of urbanization, the conclusions of this paper will be of great significance to transition countries.

## Methodology

### Econometric model

With the aim of investigating the health effects of energy poverty, this study employed the ordinary least squares model (OLS) and the Probit model.


(1) $$\begin{array}{l} health_{i}=\alpha_{0}+\alpha_{1}poverty+\alpha_{2}cons+\varepsilon_{1} \end{array}$$

Where, the dependent variables are denoted by *health*_*i*_*,i* = 1, 2, 3, corresponding to *respiratory, hospitalization* and *expenditure*, respectively; *poverty* is energy poverty; is a set of control variables; *α*_0_ is the constant term; and *ε*_1_ is the corresponding error term.

### Data

All the data was derived from the CFPS carried out by the Institute of Social Science Survey at Peking University in 2018. This survey is recognized as nationally representative which covers 14,223 families and 32,669 individuals from 25 out of 33 provinces and which is regarded as one of the richest micro-survey data in China. It collects detailed information about accessibility to modern energy. Moreover, the questionnaire also cover issues concerning respondent's health including diseases, treatment and healthcare expenditure, confirming that the data can shed light on the Chinese residents' energy poverty and health status. Additionally, the analysis objects for this research are limited to individuals over the age of 18. We precisely match the data of energy poverty at the household level with the data at the individual level by household numbers and get 27,407 valid samples.

### Variables

We use objective health to assess people's health status. To this end, respiratory diseases, hospitalization and healthcare expenditure are considered as dependent variables building on Lin et al. (43) and Bukari et al. (59). Respiratory diseases are direct outcome of energy poverty and hospitalization and healthcare expenditure can reveal the extent of illness (43, 59). We set respiratory diseases as a binary variable that equals one if the respondent has suffered from bronchitis or asthma in the past 6 months and zero if otherwise. Hospitalization is a binary variable that equals one if the respondent has hospitalized for illness during the last 12 months and zero if otherwise. Healthcare expenditure is evaluated by the money paid for illness during the last 12 months.

Our independent variable is energy poverty. It is a binary variable that equals one if the respondent's family relies on solid fuels to cook and zero if otherwise. Previous literature mainly assessed energy poverty through accessibility, affordability and multidimensionality (6, 7, 60, 61). However, current energy poverty measures focus primarily on access to modern forms of energy in developing countries (60–65). In addition, indoor air pollution generated by using unclean fuel for cooking and heating is more closely related to health status, especially respiratory diseases. Therefore, we measure energy poverty through the lens of accessibility. Table 1 presents the definition and description of variables used in this study.

**Table 1:** Definitions of variables and summary statistics.

**Variable**	**Label**	**Definition**	**Mean**	**Std**.	**Min**	**Max**
Respiratory diseases	*Respiratory*	Have you ever had physician-diagnosed bronchitis or asthma in the past 6 months? Yes = 1, No = 0	0.05	0.22	0	1
Hospitalization	*Hospitalization*	Have you ever been hospitalized for illness in the last 12 months? Yes = 1, No = 0	0.13	0.33	0	1
Healthcare expenditure	*Expenditure*	How much did you spend on treatment during the last 12 months? (yuan: logarithm form)	4.81	3.52	0	13.21
Energy poverty	*Poverty*	Does your family rely on solid fuels such as firewood and coal to cook? Yes = 1, No = 0	0.29	0.45	0	1
Female	*Female*	Female, Yes = 1, No = 0	0.50	0.50	0	1
Age	*Age*	Age	44.33	18.65	18	96
Education	*Education*	Education status (discrete variable from 1 = illiteracy to 7 = Phd)	3.02	1.36	1	7
Number	*Number*	Number of members of a household	3.77	1.85	1	8
Income	*Income*	Household incomes per capita (yuan: logarithm from)	11.07	0.83	9.21	16.03
Size	*Size*	Area of a household dwelling (cubic meters)	130.43	94.51	6	1,000
Northern provinces	*North*	If household lived in northern China = 1, otherwise = 0	0.45	0.50	0	1
Urban carbon emission	*U-emission*	Urban carbon emissions (million tons: logarithm from)	4.09	1.78	2.24	6.81
Rural Carbon emission	*R-emission*	Rural carbon emissions (million tons: logarithm from)	3.21	1.25	1.12	4.36
Housework time	*Time*	How many hours per day do you spend on housework?	0.52	1.35	0	18
Poverty ratio	*Ratio*	The average provincial share of people in energy poverty	28.85	16.85	0	62.14

## Main results

### Descriptive statistics

#### Energy poverty

**Figure 1:**
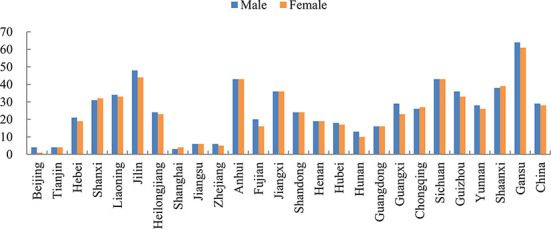
Distribution of energy poverty.

According to Figure 1, the distribution of poverty incidence varies in provinces. The average ratio of energy poverty in China is 29%. However, energy poverty ratios of Anhui, Gansu, Guizhou, Jiangxi, Jilin, Liaoning, Shanxi, Shaanxi and Sichuan are higher than 29%, which indicates that energy poverty scenarios of the above-mentioned ten provinces are severer than other provinces. Moreover, Figure 1 also shows that the incidences in eastern provinces (i.e., Beijing, Tianjin, Hebei, Liaoning, Shanghai, Jiangsu, Zhejiang, Fujian, Shandong, Guangdong) are lower than those in central (i.e., Shanxi, Jilin, Heilongjiang, Anhui, Jiangxi, Henan, Hubei, Hunan) and western provinces (i.e., Chongqing, Sichuan, Shaanxi, Yunnan, Guizhou, Gansu, Guangxi). Specifically, the average incidences are 17.97, 25.06, and 46.28%, respectively. Energy poverty and socio-economic development are highly correlated. Socio-economic development in eastern provinces is better than the latter two, accompanied by a more comprehensive clean energy grid system. Furthermore, high income ensures that residents in the eastern provinces have enough money to support energy consumption.

#### Gender disparities

**Figure 2:**
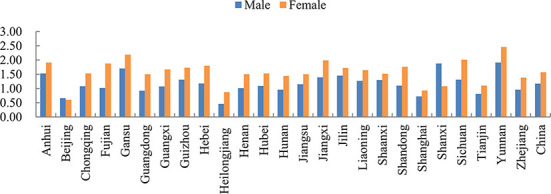
Gender difference of housework time.

Figure 1 also illustrates that there is no remarkable difference in the occurrence of energy poverty between males and females. The average energy poverty incidence of males and females are 29 and 28%, respectively, suggesting that males and females almost suffer from the same extent of energy poverty. However, as shown in Figure 2, women spend more time on housework than men, which prevails in China, and the average housework time of women is 1.57 h compared with 1.17 h of men, indicating that housework division remains unequal in Chinese family—women tend to spend more time on domestic activities like cooking and cleaning. Above all, women are prone to spend more time on housework while they suffer less from energy poverty than men, which may lead to gender inequality in terms of health impacts of energy poverty.

#### Energy poverty and health

**Figure 3:**
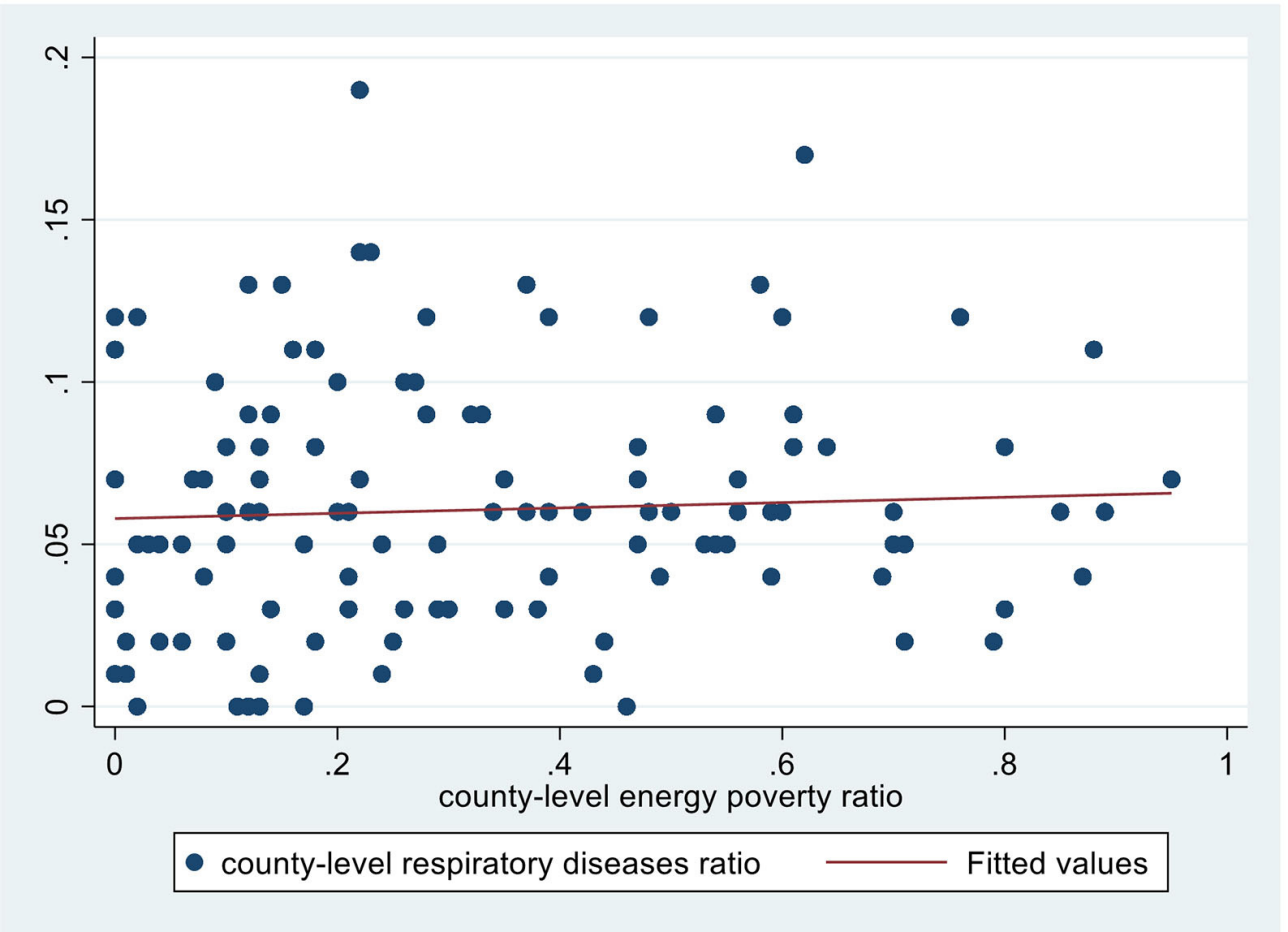
Energy poverty and respiratory diseases.

**Figure 4:**
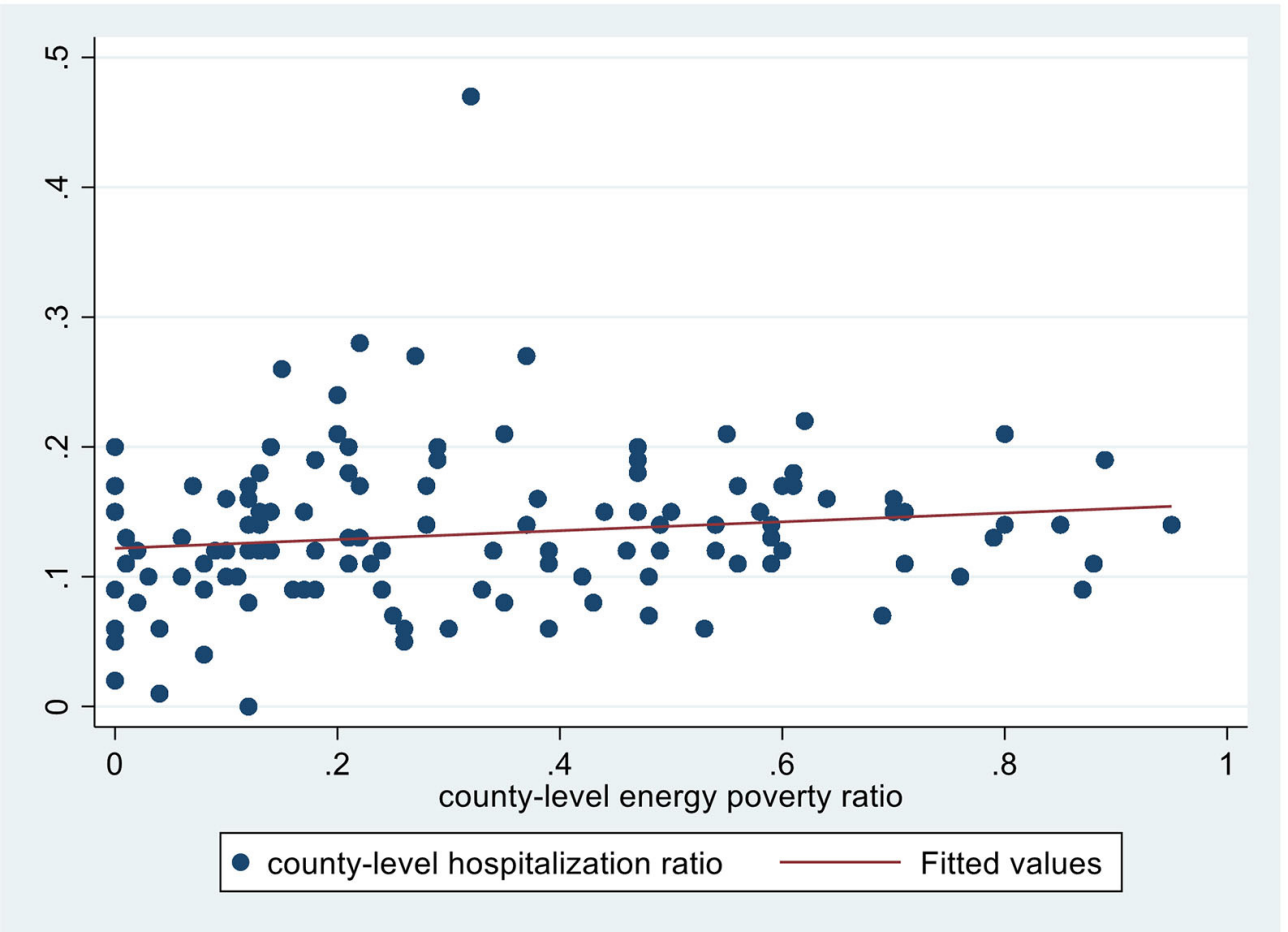
Energy poverty and hospitalization.

**Figure 5:**
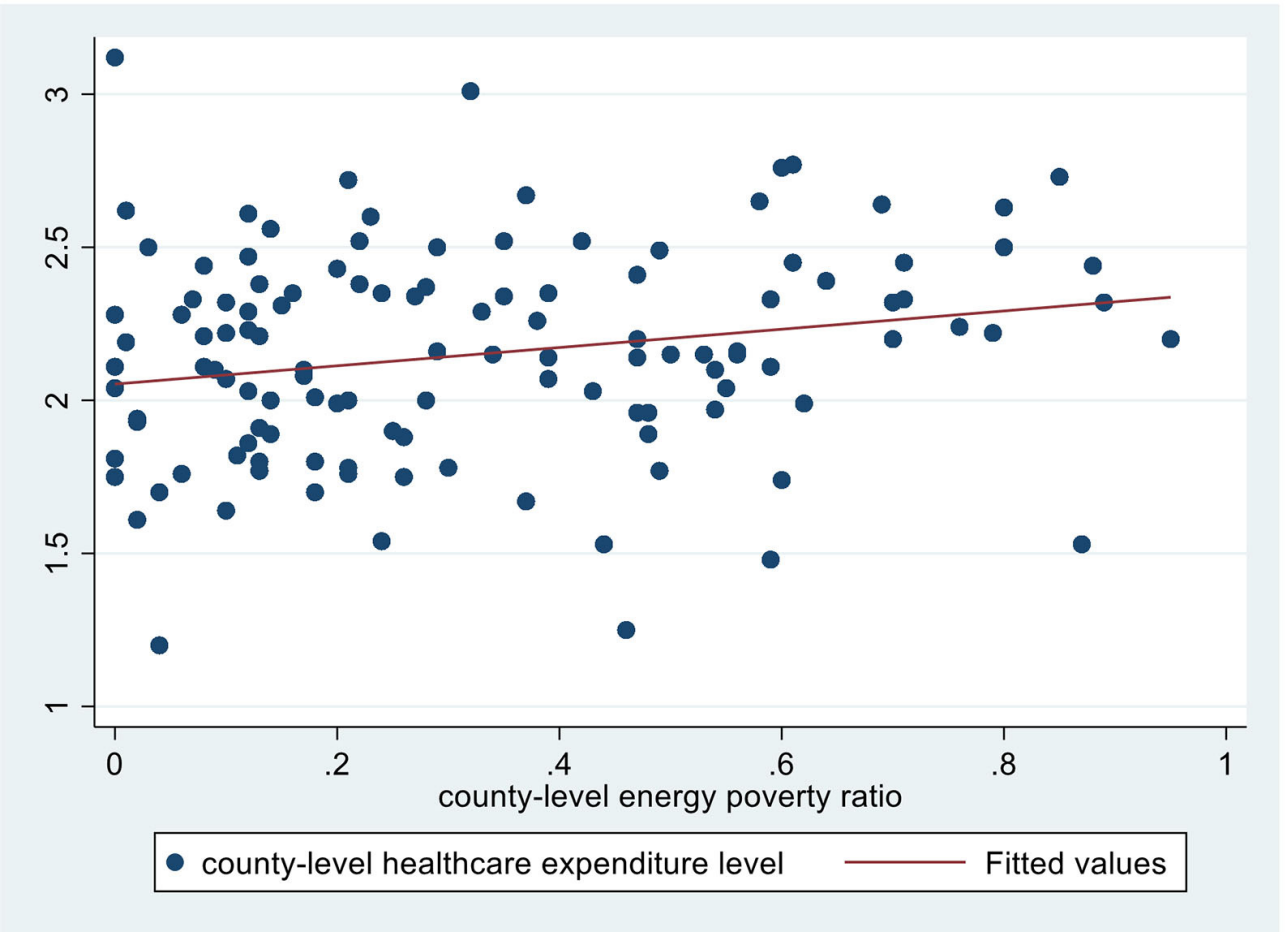
Energy poverty and healthcare expenditure.

Figures 3–5 depict the adverse health effects of energy poverty. We calculated the energy poverty ratio, incidence of respiratory diseases, hospitalization rate and average healthcare expenditure at the county level, and drew the scatter distribution and regression line between the energy poverty ratio and the latter three. As shown in Figures 3–5, all the three regression lines are inclined upward, suggesting that there is a positive nexus between energy poverty and respiratory diseases, hospitalization and healthcare expenditure. Furthermore, we also examined the Pearson correlation relationship between energy poverty and three indicators of health status. The regression coefficients were significant at magnitudes of 0.06, 0.18, and 0.24, respectively, suggesting that energy deprivation could increase the incidence of respiratory diseases and hospitalization, further increasing their healthcare expenditure.

### Baseline regression

The baseline regression results are reported in Table 2. This paper first employed OLS method to regress energy poverty and healthcare expenditure and used Probit method to examine the effects of energy poverty on respiratory diseases and hospitalization. Columns (1) and (2) present the estimation results using respiratory as explained variable. The regression coefficients of poverty are significant at magnitudes of 0.13 and 0.11, indicating that energy poverty can increase the risk of respiratory diseases. As reported in columns (3) and (4), the regression coefficients of poverty on hospitalization are significant 0.10 and 0.06, suggesting that energy poverty increases the likelihood of hospitalization. As shown in columns (5) and (6), the regression coefficients of poverty on expenditure are 0.36 and 0.24, both significant, demonstrating that the energy poor spend more money on healthcare than those not suffering from energy poverty. Due to inaccessibility to clean energy, energy-poor people are forced to use coal, firewood and other solid fuels as their primary cooking fuels, which produces pollutants and endangers human health. After inhaling these pollutants, their respiratory tract could be directly damaged and they easily suffer from bronchitis, asthma and other diseases. Consequently, the likelihood of hospitalization and the healthcare expenditure would increase.

**Table 2:** Energy poverty and health status.

**Variable**	**(1) *Respiratory***	**(2) *Respiratory***	**(4) *Hospitalization***	**(5) *Hospitalization***	**(7) *Expenditure***	**(8) *Expenditure***
*Poverty*	0.13^**^ (4.77)	0.11^**^ (3.54)	0.10^**^ (4.51)	0.05^**^ (2.46)	0.36^**^ (7.53)	0.24^**^ (4.90)
*Age*		0.02^**^ (21.73)		0.01^**^ (19.67)		0.04^**^ (22.00)
*Education*		0.06^**^ (4.28)		−0.06^**^ (−5.56)		−0.05^**^ (−2.06)
*Number*		−0.08e^−2^ (−0.10)		0.01e^−2^ (0.48)		−0.02 (−0.25)
*Size*		−0.06e^−4^ (−0.43)		0.01e^−3^ (1.14)		0.03e^−3^ (0.77)
*Income*		0.04 (0.52)		0.04^**^ (2.83)		0.01^**^ (4.60)
*North*		0.18^**^ (6.36)		0.04^*^ (1.90)		0.14^**^ (3.01)
*Constant*	−1.66^**^ (−107.81)	−2.50^**^ (−11.71)	−1.17^**^ (−100.15)	−1.42^**^ (−8.81)	4.74^**^ (176.85)	3.00^**^ (9.60)
*Pseudo R^2^/R^2^*	0.02e^−1^	0.07	0.01 e^−1^	0.06	0.08 e^−2^	0.05
*Obs*	27,047	23,910	27,048	23,911	24,891	22,127

* ,

** , and

*** respectively indicate significance at the level of 10, 5, and 1%, *t* value in parentheses.

The estimation results of control variables are also presented in Table 2, age has significant positive effects on respiratory diseases, hospitalization and healthcare expenditure, respectively. Considering that nearly 90% of the samples are adults, their physical functions are prone to decline and suffer from diseases like bronchitis or asthma with age, with more frequent hospitalization and medical expenditure. Education has positive effects on respiratory diseases with the 1% level of significance and negative effects on hospitalization and healthcare expenditure significant at the 1% and 5% level, respectively. In order to get a higher education qualification, people need to work hard indoors for a long time and spend less time breathing fresh air outdoors, therefore, their respiratory tract systems are more likely to be infected. However, higher education attainments ensure that people have better knowledge of medicine and health and more reasonable ways of life and work, which would lower the incidence of hospitalization and decrease the healthcare expenditure. Income has significant positive effects on hospitalization and healthcare expenditure, respectively. Richer people tend to receive better treatment when they get sick, thereby increasing hospitalization and healthcare expenditure. People in the north tend to experience higher incidences of respiratory diseases and hospitalization. As a result, they would spend more on healthcare, which is consistent with the conclusion of Zhang et al. (65). According to Ye and Chen (66), major pollutants, such as SO_2_ and NO_2_, are higher in the north than in the south, especially when residential heating caused more serious smog pollution in the north in winter.

### Endogeneity

Given the potential endogenous problems caused by unobserved variables and other factors, we selected the average provincial share of people in energy poverty (i.e., ratio) as an instrumental variable (IV) drawing on Zhang et al. (65). For one thing, the residents in the same province have few clear-cut distinctions in terms public facility construction level and so on. Hence, household-level energy poverty is strongly related to incidence at the provincial level. For another, people's health is affected by many factors like income, education and age, which has nothing to do with provincial-level energy poverty ratio. Above all, it is considerably reasonable to select the provincial-level energy poverty incidence as IV.

We used instrumental variable probit (Ivprobit) method to regress respiratory and hospitalization because both of them are binary variables; while we employed two-stage least squares (2SLS) method to expenditure because it is a continuous variable. The results of regressions are reported in Table 3. Columns (4) to (6) report the regression results of poverty on respiratory, hospitalization and expenditure respectively. The results show that the Wald chi (2) values of models (1) to (3) are 13.44, 76.63, and 82.84, respectively, with the 1% level of significance, rejecting the null hypothesis that there is an endogenous problem of energy poverty. Moreover, the values of the t statistic of IV are 56.84, 56.84, and 52.69 respectively, indicating that IV is exogenous. In addition, the values of the F statistic estimated in the first stage are 748.12, 748.22, and 2,766.18 respectively, confirming no weak IV problem. The above results reveal that it is reasonable to select the provincial-level incidence of energy poverty as an instrumental variable.

**Table 3:** Energy poverty and health status: Ivprobit & 2SLS.

**Variable**	***First stage regression***			***Second stage regression***		
	**(1) *Poverty***	**(2) *Poverty***	**(3) *Poverty***	**(4) *Respiratory***	**(5) *Hospitalization***	**(6) *Expenditure***
*Ratio*	0.96e^−2^^***^ (56.84)	0.76^***^ (56.84)	0.95e^−2^^***^ (52.69)			
*Poverty*				0.49^***^ (5.12)	0.63^***^ (8.96)	1.56^***^ (10.43)
*Control*	*Yes*	*Yes*	*Yes*	*Yes*	*Yes*	*Yes*
*F value of First stage*	748.12^***^	748.22^***^	2,766.18^***^			
*t value of IV*	56.84^***^	56.84^***^	52.69^***^			
*Wu-Hausman/Wald chi(2)*				13.44^***^	76.63^***^	82.84^***^
*R^2^*	0.15	0.15	0.03	0.15	0.15	0.03
*Obs*	25,318	25,319	23,481	25,318	25,319	23,481

* ,

** , and

*** respectively indicate significance at the level of 10, 5, and 1%, *t* value in parentheses.

After introducing the IV, the coefficients of poverty on respiratory, hospitalization and expenditure are all significant at magnitudes of 0.49, 0.63, and 1.56, respectively, suggesting that energy poverty still hamper residents' health in consideration of endogeneity. Meanwhile, the coefficients of Ivprobit and 2SLS regression are higher in comparison with Probit and OLS regression, indicating that the effects may be underestimated without considering endogeneity, which is consistent with the conclusion of Phoumina and Kimurab (67).

### Moderating effect

As stated in the Introduction, air pollution induced by cooking, heating and other household activities is the dominant reason for residents' health hazards. Therefore, if the energy poor engage in housework for a relatively long time, it will undoubtedly increase their frequency and quantity of toxic gas and particle inhalation, and health status will be harmed more severely. Hence, it is reasonable to assume that housework time plays a moderating role in the effect of energy poverty on residents' health. In order to test this hypothesis, this paper sets up the following econometric model


(2) $$\begin{array}{l} health_{i}=\beta_{0}+\beta_{1}poverty+\beta_{2}time+\beta_{3}poverty*time\\ \;+\beta_{4}cons+\varepsilon_{2} \end{array}$$

Where, the dependent variables are denoted by *health*_*i*_, *i* = 1, 2, 3, corresponding to *respiratory, hospitalization* and *expenditure*, respectively; *poverty* is energy poverty; *time* is housework time; poverty^*^time is the cross term of energy poverty and housework time.

As presented in Table 4, columns (2), (4) and (6) report the regression results of health status after introducing the cross term. Firstly, coefficients of cross term in columns (2) and (4) are 0.10 and 0.14, both significant, suggesting that there is a moderating effect of housework time on the nexus between energy poverty on *respiratory* and *hospitalization*. Moreover, the coefficients of *poverty* in in columns (2) and (4) are 0.42 and 0.57, both significant at the level of 1%, suggesting that the moderating effect is positive. In other words, housework time can increase the likelihood of residents suffering from respiratory diseases and hospitalization in energy-poor families. For individuals suffering from energy poverty, the longer they spend on housework like cooking and heating, the more toxic gases and particles they inhale, the severer damage their respiratory tracts suffer. In addition, hazards to respiratory tracts will endanger other parts of body. Hospitalizations are necessary if the condition worsens over time.

**Table 4:** Moderating effect test.

**Variable**	**(1) *Respiratory***	**(2) *Respiratory***	**(3) *Hospitalization***	**(4) *Hospitalization***	**(5) *Expenditure***	**(6) *Expenditure***
*Poverty*	0.49^***^ (5.12)	0.42^***^ (4.21)	0.63^***^ (8.96)	0.57^***^ (7.64)	1.56^***^ (10.43)	1.67^***^ (10.65)
Poverty^*^time		0.10^*^ (1.86)		0.14^***^ (3.22)		−0.04 (−0.42)
*Control*	*Yes*	*Yes*	*Yes*	*Yes*	*Yes*	*Yes*
*R^2^*	0.15	0.31	0.23	0.31	0.03	0.31
*Obs*	25,318	25,309	25,319	25,310	23,481	23,344

* ,

** , and

*** respectively indicate significance at the level of 10, 5, and 1%, *t* value in parentheses.

### Gender inequality

As stated in section Moderating effect, the health impacts of energy poverty are moderated by housework time. Considering traditional gender role—men being the breadwinners and women being the homemakers—in the division of housework in China, the time of females engaging in housework is much longer than that of males, which may lead women to suffer from more indoor air pollution and severer impacts on health status. Therefore, we adopted the following equation to examine whether the gender inequality exists in the health effects of energy poverty.


(3) $$\begin{array}{l} health_{i}=\gamma_{0}+\gamma_{1}poverty+\gamma_{2}female+\gamma_{3}poverty*female\\ \;+\gamma_{4}cons+\varepsilon_{3} \end{array}$$

Where, the dependent variables are denoted by *health*_*i*_, *i* = 1, 2, 3, corresponding to *respiratory, hospitalization* and *expenditure*, respectively; *poverty* is energy poverty, poverty^*^female is the cross term of energy poverty and female. The results are shown in Table 5.

**Table 5:** Gender inequality in the impacts of energy poverty on health: Urban-rural disparities.

**Variable**	***Total***			***Rural***			***Urban***		
	**(1)** ***Respiratory***	**(2)** ***Hospitalization***	**(3)** ***Expenditure***	**(4)** ***Respiratory***	**(5)** ***Hospitalization***	**(6)** ***Expenditure***	**(7)** ***Respiratory***	**(8)** ***Hospitalization***	**(9)** ***Expenditure***
*Poverty*	0.32^***^ (2.64)	0.41^***^ (4.37)	1.31^***^ (6.59)	0.24^*^ (1.97)	0.30^**^ (2.40)	1.53^***^ (6.00)	0.72^**^ (1.99)	1.04^***^ (5.85)	1.71^**^ (2.73)
Poverty*female	0.32^**^ (1.97)	0.44^***^ (3.52)	0.62^***^ (2.34)	0.10^**^ (2.50)	0.64^***^ (3.97)	0.61^*^ (1.89)	0.65 (1.18)	0.48 (1.21)	0.63 (0.67)
*Control*	*Yes*	*Yes*	*Yes*	*Yes*	*Yes*	*Yes*	*Yes*	*Yes*	*Yes*
*R^2^*	0.28	0.28	0.29	0.39	0.40	0.39	0.11	0.11	0.11
*Obs*	25,318	25,319	23,481	12,743	12,743	11,672	12,433	12,434	11,672

* ,

** , and

*** respectively indicate significance at the level of 10, 5, and 1%, *t* value in parentheses.

We introduced the cross item poverty^*^female to regress with *respiratory, hospitalization* as well as *expenditure* respectively. The results are reported in Table 5. Columns (1) to (3) report the regression results of the health status. The coefficients of *poverty* are all significant at magnitudes of 0.32, 0.41, and 1.31, while the coefficients of poverty^*^female are all significant at magnitudes of 0.32, 0.44, and 0.62. The results reveal that female plays a positive moderating role in the nexus between energy poverty and the three proxies of health. In other words, females who suffer from energy poverty have a higher tendency to experience respiratory diseases and hospitalization as well as spend more on healthcare. Females are traditionally considered as the homemakers and devote more to the domestic work such as cooking and heating. On the contrary, males are usually considered as the breadwinners and pay less attention to homecare. Consequently, the frequency and quantity of toxic gases inhaled by females due to cooking, heating and other chores are higher than that of males, which results in the gender inequality in terms of health impacts of energy poverty.

Owing to urban-rural dual structure, urban women can get more job opportunities and work outside compared to rural women. Both husbands and wives can become breadwinner in urban families, which may decrease the housework time of females and change the traditional division of labor within the family. Therefore, we classified the samples into urban subgroup and rural subgroup to examine the heterogeneity across areas. Additionally, given that air quality disparity between rural and urban areas, we introduce carbon emission as control variables. Columns (4) to (6) present the regression results of rural residents' health. The coefficients of poverty and poverty^*^female are significantly positive, suggesting that there is a positive moderating effect of female on the impacts of energy poverty on hospitalization and healthcare expenditure. In other words, gender inequality in terms of health impacts of energy poverty do exist in rural areas. Females in rural areas are considered as the homemakers and devote more to the domestic work such as cooking and heating, they are more likely to be impacted by energy poverty and experience higher rate of hospitalization as well as spend more on healthcare. However, as shown in columns (7) to (9), the coefficients of poverty are significantly positive while the coefficients of poverty^*^female are not significant, suggesting that this is no moderating effect of female on the nexus between energy poverty and health. In other words, gender inequality in terms of health impacts of energy poverty do not exist in urban areas. Compared with rural women, the status of urban women has been promoted to a large extent with the improvement of their economic status. Accordingly, intra-household division of labor becomes relatively equal in urban areas. Ultimately, gender gaps in the health impacts of energy poverty will be narrowed (52, 68).

We further classified the samples into young subgroup (from 18 to 39 years old), middle-age subgroup (from 40 to 59 years old) and old subgroup (over 60 years old) to examine the heterogeneity across ages. The results are reported in Table 6. Columns (1) to (3) present the regression results of young residents' health, the coefficients of poverty^*^female are not significant, indicating that there is no moderating effect of female on health. Columns (4) to (6) present the regression results of middle age residents' health. The coefficients of poverty and poverty^*^female in columns (5) and (6) are significantly positive, suggesting that there is a positive moderating effect of female on the impacts of energy poverty on hospitalization and healthcare expenditure. The results of the old subgroup are similar to those of the middle-age group. Firstly, more and more young women become breadwinners with the rapid development of economy due to reform and opening policy since 1978, their housework time may decrease after getting equal economic status. In addition, development of economy also accelerates the progress of social concepts of gender equality, calling upon men to share housework with women.

**Table 6:** Gender inequality in the impacts of energy poverty on health: Age disparities.

**Variable**	***Young***			***Middle-age***			***Old***		
	**(1)** ***Respiratory***	**(2)** ***Hospitalization***	**(3)** ***Expenditure***	**(4)** ***Respiratory***	**(5)** ***Hospitalization***	**(6)** ***Expenditure***	**(7)** ***Respiratory***	**(8)** ***Hospitalization***	**(9)** ***Expenditure***
*Poverty*	0.13 (0.33)	0.08 (0.30)	0.34 (0.82)	0.15 (0.68)	0.45^**^ (2.51)	2.08^***^ (6.31)	0.36^**^ (2.23)	0.43^***^ (3.13)	0.96^***^ (2.96)
Poverty^*^female	0.38 (0.71)	0.50 (1.34)	0.68 (1.17)	0.47 (1.59)	0.50^**^ (2.24)	0.71^*^ (1.69)	0.31 (1.42)	0.52^***^ (2.86)	0.80^*^ (1.91)
*Control*	*Yes*	*Yes*	*Yes*	*Yes*	*Yes*	*Yes*	*Yes*	*Yes*	*Yes*
*R^2^*	0.29	0.28	0.20	0.18	0.29	0.29	0.27	0.11	0.27
*Obs*	3,700	3,700	3,700	8,290	8,290	8,290	10,685	10,686	10,685

* ,

** , and

*** respectively indicate significance at the level of 10, 5, and 1%, *t* value in parentheses.

### Historical origins

Section Gender inequality reveals gender inequality in the health impacts of energy poverty. Given that status inequality is the dominant reason for gender differences in housework time, we further examined the nexus between status inequality and housework time. We adopted the following model to identify the effects of status inequality on housework time.


(4) $$\begin{array}{l} time=\phi_{0}+\phi_{1}inequality+\phi_{2}cons+\varepsilon_{4} \end{array}$$

Where, *time* is housework time; *inequality* is status inequality. Additionally, in view of the significantly negative relationship between housework time and job, we add variable *job* into control variables.

Because of the 9-year compulsory education system in China, parents do not have to pay for their children's education until high school. On the basis of Li et al. (69), we selected the gender ratio of high school enrollment rate as the proxy of status inequality to regress the housework time of males and females, respectively. As presented in Table 7, columns (1) and (4) report the regression results of total sample. The coefficient of *inequality* is not significant in consideration of control variables, indicating that status inequality doesn't increase the housework time in the whole society. As presented in columns (2) and (5), the coefficients of female subsample are significant in consideration of control variables, suggesting that status inequality increases the housework time of females. Columns (3) and (6) report the regression results of male subsample, the coefficients are not significant no matter whether control variables are introduced or not, suggesting that status inequality doesn't increase the housework time of males. Our findings point out that status inequality increases the housework time of females significantly, but it does not have the same effect on males. On the one hand, status inequality leads to disproportionate division of housework time, and women take more responsibility for domestic affairs owing to the relatively low economic status. On the other hand, the uneven intra-household division of labor may be reinforced by social customs and cultural norms.

**Table 7:** Status inequality and housework time.

**Variable**	**(1) *Total***	**(2) *Female***	**(3) *Male***	**(4) *Total***	**(5) *Female***	**(6) *Male***
*Inequality*	−0.08^*^ (−1.69)	−0.02 (−0.29)	−0.06 (−1.22)	0.02 (0.37)	0.12^*^ (1.68)	−0.06 (−1.33)
*Control*	*No*	*No*	*No*	*Yes*	*Yes*	*Yes*
*R^2^*	0.23	0.31	0.29	0.40	0.46	0.34
*Obs*	27,090	13,590	13,500	25,361	12,713	12,648

* ,

** , and

*** respectively indicate significance at the level of 10, 5, and 1%, *t* value in parentheses.

### Robustness checks

We select respiratory, hospitalization and expenditure as dependent variables in above empirical analysis, and all measured health from the perspective of objective health. As a comparison, we use subjective health to assess health status in this section (65). Specially, we use self-reported health (How would you rate your health status? discrete variable from 1 = poor to 5 = excellent) and interviewer-reported health (Respondent's health status observed by interviewer, discrete variable from 1 = very poor to 7 = very good) as dependent variables which measure health from the perspective of subjective to conduct a robustness test. The regression results are depicted in Table 8. Column (1) to (8) report the results of baseline regression, endogeneity, moderating effect and gender inequality. The findings are consistent with previous findings, indicating that the empirical results are robust in this paper.

**Table 8:** Robustness checks using the alternative dependent variable.

**Variable**	***Self-reported health***					***Interviewer-reported health***				
	**(1) *Health***	**(2) *Health***	**(3) *Health (rural)***	**(4) *Health (urban)***	**(5) *Health***	**(6) *Health***	**(7) *Health***	**(8) *Health (rural)***	**(9) *Health (urban)***	**(10) *Health***
*Poverty*	−0.28^***^ (−6.02)	−0.27^***^ (−5.48)	−0.50^***^ (−4.61)	−0.78^***^ (2.77)	−0.19^***^ (−3.23)	−0.63^***^ (−11.29)	−0.60^***^ (−10.30)	−0.31^***^ (−3.37)	−0.52^***^ (−4.15)	−0.46^***^ (−6.39)
Poverty^*^Time		−0.07^**^(−2.22)					−0.10^**^(−2.52)			
Poverty*female			−0.40^***^ (−2.96)	−0.29 (−0.97)	−0.22^***^ (−2.62)			−0.19^***^ (−5.16)	−0.16 (−0.70)	−0.36^***^ (−3.60)
*Control*	*Yes*	*Yes*	*Yes*	*Yes*	*Yes*	*Yes*	*Yes*	*Yes*	*Yes*	*Yes*
*F value of First stage*	838.63^***^					719.50^***^				
*t value of IV* (*ratio*)	55.34^***^					51.23^***^				
*Wu-Hausman/ Wald chi(2)*	15.27^***^					28.94^***^				
*R^2^*	0.14	0.14	0.12	0.12	0.14	0.13	0.13	0.15	0.14	0.13
*Obs*	25,316	25,307	10,728	10,093	25,316	20,887	20,881	12,423	12,580	20,887

* ,

** , and

*** respectively indicate significance at the level of 10, 5, and 1%, *t* value in parentheses.

## Conclusion and policy implication

Using solid fuels for household chores will generate indoor air pollution and threaten the health of residents, while the disproportionate division of labor within the family may lead to gender inequality in the health hazards of energy poverty. Using CFPS 2018 dataset, we studied the gender inequality in the health impacts of energy poverty. First, we examined the causality between energy poverty and health status and the moderating effects of housework time. Then, we analyzed the gender inequality in health impacts of energy poverty and heterogeneous effects of energy poverty on health across sectors and ages. Finally, we further investigated the nexus between social status inequality and housework time.

Our results reveal that energy poverty increases the odds of respiratory diseases, hospitalization as well as the health expenditure of the residents. In addition, housework time plays a moderating role in the causality between energy poverty and health. The more time energy-poor groups spend on housework, the worse their health will be. As a result, women are more susceptible to indoor air pollution since they usually devote more to domestic chores than men. However, the gender inequality of energy poverty on health is weakening with urbanization. Further analyses indicate that the root of gender inequality in the health impacts of energy poverty is status inequality.

Although considerable literature focused on gender inequality in health impacts of energy poverty, this paper distinguishes itself from existing literature in following ways. Firstly, compared with previous studies (24, 44, 65, 70–72), we empirically examined the gender inequality effects on the impacts of energy poverty on health, and the trend in the context of urbanization, development of economy and progress of social concepts. Moreover, our analysis clarified the mechanism how energy poverty leads to gender inequality of health impacts while the moderating role of housework time has been devoted to little attention in existing literature (4, 5, 67). In addition, our findings further pointed out that above phenomenon is caused by social structures, which has been ignored in previous studies (27, 73).

Findings from this paper will contribute to policy formulation. In the context of the developing countries like China, the study suggests that policymakers should attach great importance to energy poverty alleviation. On the one hand, the government should invest more in modern energy infrastructure systems and clean energy technology to ensure that the energy-poor households could have access to modern energy. These initiatives should pay more attention to rural and underdeveloped areas since most of those areas are off-grid. On the other hand, subsidies on clean energy and related appliance energy consumption for energy-poor households should also be taken into consideration.

The government should also improve the kitchen ventilation system of energy-poor families because cooking accounts for most of the housework time and plays a vital role in indoor air pollution. It is necessary to provide the energy poor families with cleaner stoves, chimneys, smoke lampblack machines and other ventilation equipment by subsiding so as to decrease the frequency and quantity of toxic gas and particle inhalation and thus alleviate the hazards of indoor air pollution.

Moreover, it is important to eliminate the urban-rural dual structure. The breakdown of the urban-rural dual structure will be conducive to labor migration from rural to urban areas and providing more job opportunities for rural women. As a result, the social status of rural females will be promoted with the improvement of economic status, further leading to the relatively even intra-household division of labor and facilitating the gender equality.

In addition, energy-poor households are likely to have lower health status, which may force them to spend more on healthcare and push them into the vicious circle of poverty. To avoid this trend, social security system for energy-poor households through medical insurance should be enhanced. Diseases related to energy poverty, such as chronic obstructive pulmonary diseases (COPDs) and cardiovascular diseases should be incorporated in the medical insurance. Moreover, the government should also focus on screening and treating these diseases for housewives as they are more likely to expose to indoor air pollution.

Finally, the government should also legislate to ensure gender equality between men and women in the whole society. In addition, the role of women's rights protection organization and mass media should not be neglected. Social norms that men being the breadwinners and women being the homemakers in the division of housework can probably be changed in a subtle way as time passes by.

## Data availability statement

The original contributions presented in the study are included in the article/supplementary material, further inquiries can be directed to the corresponding author.

## Author contributions

ZZ: conceptualization, methodology, software, and resources. YL: investigation and writing—original draft. XM: data curation and software. HY: visualization, validation, supervision, and writing—review and editing. All authors contributed to the study conception and design.
